# ⁠⁠Functional Foods Alleviate Behavioral Alterations and Improve GABAergic System Regulating TLR‐4/NF‐κB Axis in Valproic‐Induced Autism

**DOI:** 10.1002/brb3.70591

**Published:** 2025-06-04

**Authors:** Francesco Molinari, Roberta Fusco, Rosalba Siracusa, Ramona D'Amico, Daniela Impellizzeri, Ali S. Abdelhameed, Tilman Fritsch, Ursula M. Jacob, Salvatore Cuzzocrea, Vittorio Calabrese, Rosanna Di Paola, Marika Cordaro

**Affiliations:** ^1^ Department of Veterinary Sciences University of Messina Messina Italy; ^2^ Department of Chemical, Biological, Pharmaceutical, and Environmental Sciences University of Messina Messina Italy; ^3^ Department of Pharmaceutical Chemistry, College of Pharmacy King Saud University Riyadh Kingdom of Saudi Arabia; ^4^ NAM‐Institute Salzburg Austria; ^5^ System Biologie AG Wollerau Switzerland; ^6^ Link Campus University Rome Italy; ^7^ Department of Biomedical and Biotechnological Sciences University of Catania Catania Italy; ^8^ Department of Biomedical Dental and Morphological and Functional Imaging, University of Messina Messina Italy

**Keywords:** autism, behaviors, GABA, neuroinflammation, TLR‐4, valproic acid

## Abstract

**Scope:**

Valproic acid (VPA) postnatal exposure in mice results in behavioral impairment, aberrant sensitivity to sensory stimuli, and self‐harming behavior, hallmarks of autism. According to previous reports, *Coriolus versicolor* (CV) has a protective effect on the brain. The goal of the current investigation was to assess how CV affected the neurobehavioral and metabolic changes caused by VPA in mice.

**Methods and Results:**

Mice pups were injected with VPA at 14 days of age and orally administered CV at a dose of 200 mg/kg daily from 14 to 40 days of age. Mice pups were placed through behavioral tests during the trial to evaluate motor skill growth, nociceptive response, locomotion, anxiety, and cognition. Following behavioral testing, mice were killed, and the brain was removed and subjected to biochemical analyses (glutathione, malondialdehyde, and nitric oxide) and histopathological analysis. Additionally, to further investigate the role of the TLR‐4/Myd88/NF‐κB signaling pathway, we examined the modulation of this pathway and the alteration in gamma‐amino butyric acid (GABA) production using Western blot analysis.

**Conclusion:**

According to our research, CV daily administration greatly reduced behavioral alteration, reversed the disorganization of the cerebellum and hippocampus, and significantly improved the VPA‐induced neuroinflammation via the TLR‐4/Myd88/NF‐κB signaling cascade.

## Graphical abstract

Dietary supplementation with *Coriolus versicolor* ameliorate neuroinflammation VPA‐induced.

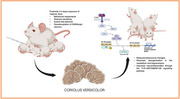

## 1 Introduction

Autism is a neurodevelopmental disorder characterized by self‐harming behaviors, stereotypic conduct, poor speech, impaired social behaviors, and poor communication skills (Pragnya et al. 2014). Increased oxidative stress, hyperserotonemia, and loss of Purkinje cell integrity in the cerebellum are the main pathology findings of autism (Chaste and Leboyer 2012; Hranilovic et al. 2009; Hranilovic et al. 2007; Kern and Jones 2006; Liu et al. 2022). The condition typically manifests before the age of three. Multiple factors, including genetic anomalies, environmental insults, and social factors, contribute to the development of autism, even if the etiology is still unknown (Chaste and Leboyer 2012). Autism is caused by early exposure to chemicals, medicines, or environmental irritants during crucial developmental stages. Chemicals such as mercury, ethanol, and thalidomide cause the production of reactive oxygen species (ROS), which are responsible for deficiencies in the cerebellum, limbic system, and brain development (Pham et al. 2022). Antiepileptic medication valproic acid (VPA) is well recognized to produce fetal valproate syndrome, which manifests as delays in behavioral development, language and communication deficiencies, stereotypic behaviors, and hyperexcitability (Markram et al. 2008; Santos de Oliveira et al. 2006). In particular, two different models based on VPA exposure, postnatally or prenatally, were often used to induce autistic‐like symptoms. These two models are very similar, but in the prenatal model (4.3%), it was much lower compared to the postnatal model (22.7%) (Elnahas et al. 2021). The VPA‐induced animal model is that administering VPA during the first 14 days of life interrupts neurodevelopmental processes, resulting in behavioral abnormalities resembling autism (Mony et al. 2016; Norton et al. 2020; Reynolds et al. 2012). In the cerebellum and hippocampus, neuronal migration, differentiation, myelination, synaptogenesis, and gliogenesis occur during PND (postnatal day) 14, which is a critical time (Schneider et al. 2001; Wagner et al. 2006). It has been observed that giving VPA to rodents on PND 14 results in intrusions and neurodevelopmental regressions that impair behaviors (Kern and Jones 2006; Rossignol and Frye 2014; Yochum et al. 2008). Recent studies revealed that the toll‐like receptor (TLR) signaling pathway is connected to the immunoregulatory effects of polysaccharides, and TLR4 is crucial for the stimulation of the innate immune response and the production of cytokines induced by polysaccharides (Ando et al. 2002; Han et al. 2003). The host defense system recognizes molecular structures that are shared by many infections, in part, because of a family of TLRs (Hsu et al. 2004; Yin et al. 2010). In peripheral samples and brain postmortem tissue from depressed and suicidal patients, some components of the TLR‐4 signaling pathway are upregulated. According to the “leaky gut” theory, neuropsychiatric illnesses are caused by an increase in intestinal permeability, which allows germs to move into the body and activate TLR‐4 (Garcia Bueno et al. 2016). Increased peripheral TLR‐4 expression and activity have been observed in individuals with bipolar disorder, schizophrenia, and autistic children (Enstrom et al. 2010). A change in TLR4 has also been observed to result in motor deficits in animal models of neurodegenerative diseases and traumatic brain injury (Acioglu et al. 2022; Dabi et al. 2023; Khariv et al. 2013; Koedel et al. 2007; Zou et al. 2023). The alteration of TLR4 also leads to an imbalance in the number of Purkinje cells (PCs), which are the cerebellar cortex's only output neurons. This impairs motor function because PCs oversee the control of cerebellar function, which is crucial for balance and motor coordination (Abg Abd Wahab et al. 2019). Natural remedies like green tea extract, aqueous extract of *Bacopa monnieri*, and vitamin E have been shown to prevent VPA‐induced behavioral changes in mice when used early on (Banji et al. 2011; Sandhya et al. 2012). *Coriolus versicolor* (CV) is a member of the Polyporaceae family (Chang et al. 2017), and has been utilized as a “magic herb” to encourage wellness, sturdiness, and longevity in Asian countries, particularly in China (Saleh et al. 2017; Wang et al. 2015). The use of CV extracts in integrated cancer therapy in conjunction with chemotherapy or radiotherapy has been authorized since 1987 in China and since 1977 in Japan (Chang et al. 2017; Venturella et al. 2021). CV contains a variety of bioactive compounds, such as terpenes, proteins, peptides, amino acids, purpurins, and two protein‐bound polysaccharides (the polysaccharide peptide [PSP] and the glycoprotein [PSK] krestin) (Chang et al. 2017). However, the most investigated mushroom bio‐compounds and active biological elements in mushrooms are PSP and PSK (Wang et al. 2015). Along with additional physiological effects like liver protection, system balance, antiulcer, antiaging, and learning and memory‐enhancing qualities, anticancer, anti‐inflammatory, and antiviral activities have also been noted (Cordaro et al. 2022; D'Amico et al. 2021; Habtemariam 2020; Impellizzeri et al. 2022; Jedrzejewski et al. 2020; Li et al. 2020; Pawlikowska et al. 2020; Scuto et al. 2019). It's interesting to note that CV has also been demonstrated to lessen the side effects associated with radiotherapy and chemotherapy treatments. Additionally, CV exerts neuroprotective effects in hippocampal degeneration after sepsis as well as in Alzheimer's disease (D'Amico et al. 2023; Trovato et al. 2016). With this background in mind, to further elucidate the molecular mechanisms by which CV can ameliorate autistic disorder in this paper, C57BL mice were injected with valproic acid and treated for 40 days with 200 mg/kg of CV, and the TLR4/NF‐κB pathways were evaluated.

## 2 Materials and Methods

### 2.1 Animals and Experimental Groups

The C57/BL6 mice were purchased from Envigo (Milan, Italy) and kept in a controlled environment with access to water and standard rodent chow (Teklad standard diet, purchased from Envigo). They were kept at 21 ± 1°C and 50 ± 5% humidity in a 12:12 h light:dark cycle with one female mouse per cage. The study was approved by the University of Messina Review Board for Animal Care (OPBA). Every animal experiment complies with the ARRIVE guidelines (https://arriveguidelines.org/arrive‐guidelines), EU legislation (EU Directive 2010/63), and the new Italian regulations (D.Lgs 2014/26). (P.R. 89126.51).

On PND14, male C57/BL6 mice were treated with saline (sham group) or CV (sham–CV) ([data not shown because we don't find any difference between the sham group and sham–CV] Figure S1) or sodium valproate subcutaneously (VPA group) at a dose of 400 mg/kg dissolved in sterile saline (Banji et al. 2011; Pragnya et al. 2014). CV was given orally for 26 days from PND 14 to PND 40 at a dose of 200 mg/kg dissolved in sterile saline (VPA + CV group). Mice from every treatment group were included in each litter (e.g., saline, valproate, and/or CV‐treated mice) to rule out the possibility that variations in mother care could have an impact on behavioral results. Pups in a litter were specifically color‐coded by marking their tails with a lab marker every day (red, black, or blue). CV was generously provided by Mycology Research Laboratories Ltd. (MRL, Luton, UK), and the dose was chosen on the basis of previous research (Cordaro et al. 2022; D'Amico et al. 2023; D'Amico et al. 2021; Ferreiro et al. 2018; Impellizzeri et al. 2022; Scuto et al. 2019; Trovato et al. 2016). Animals were killed under anesthesia at the conclusion of the behavioral testing, and the brain was removed for biochemical measurements or histological analysis. The study was approved by the University of Messina's animal care review board (OPBA).

### 2.2 Behavioral Investigations

Mouse pups were subjected to behavioral testing on PND40 to evaluate their motor coordination, nociceptive response, locomotion, anxiety, sociability, and cognition. All behavioral research was conducted between 9:00 a.m. and 3:00 p.m. during the light phase.

#### 2.2.1 Negative Geotaxis

Negative geotaxis is an innate vestibular response used to gauge a pup's sensorimotor proficiency and identify geogravitational stimuli (Farghaly et al. 2023). In a temperature‐controlled environment, negative geotropism was assessed by positioning the mouse with its back to the ground on a wire grid with a 45° inclination. For each trial, a maximum of 30 s of the latency to reorient and turn 180° with the head facing uphill was recorded (Pragnya et al. 2014). To increase the validity of NG experiments, two independent evaluators who were blind to study group allocation conducted and assessed the studies' outcomes (Ruhela et al. 2019).

#### 2.2.2 Nociception

Both central and peripheral processes are thought to contribute to the antinociceptive response on the hot plate (Pragnya et al. 2014). Using an Eddy's Hotplate analgesiometer (INCO Pvt. Ltd.) with an electrically heated surface, the nociceptive response of mice was assessed. The environment was kept at 55 ± 0.5°C. The animals were placed on the hot plate, and a stopwatch was used to time how long it took for them to lick their paws or jump (Ozdemir et al. 2012).

#### 2.2.3 Rotarod Test

To evaluate motor coordination and motor learning, we used a rotating rod maintained at a speed of 40 RPM (rotations per minute) to measure motor coordination (Bath and Pimentel 2017). Each mouse was placed on a rotating rod, and it was timed for 5 min to see how long it took each one to balance there (Schneider and Przewlocki 2005).

#### 2.2.4 Elevated Plus Maze Test

To assess the anxiety state as previously reported, the elevated plus maze test (EPM) was used (Norton et al. 2020; Walf and Frye 2007). The raised plus‐maze was 50 cm above the ground and had two opposing open arms and two opposing arms enclosed by opaque walls that were 15 cm high. Mice were positioned in the middle of the raised plus‐maze, facing one of the open arms, following the pretest. After a training of 10 min, it was counted how many times the mice entered each arm and how long they spent in open arms (Schneider and Przewlocki 2005; Schneider et al. 2006).

#### 2.2.5 Morris Water Maze

Hippocampal‐dependent spatial learning and memory were assessed using the Morris Water Maze (MWM) test (Siebold et al. 2020; Zhao et al. 2017). The maze was made up of a circular tub that was 21 cm high and 71 cm in circumference. The tub's interior was painted white, and it was filled halfway with water that was kept between 23°C and 26°C. White nontoxic latex paint was used to make the tub opaque. A beginning point was chosen at random from each of the four quadrants that were evenly spaced. A platform that was 8 cm in length and painted black was positioned in one quadrant of the water maze's visible platform variant. The water was only permitted to fill the platform up to 1.5 cm above the ground. An identical, white‐painted platform was situated 2 cm below the water's surface in the hidden platform. The animals were given five tries per day, and they had no more than 60 s to get to the escape platform. Throughout the trial, the hidden platform stayed in the same spot, the room was lit, and there were additional maze signals. The animal was gently led to the platform and placed there if it did not arrive in 60 s. A notable reduction in latency time as compared to the initial session was deemed indicative of successful learning. It was noted how much time was spent in the target quadrant (Pragnya et al. 2014).

#### 2.2.6 Open Field Test

The OFT was used to track locomotor activity and anxiety‐like behaviors for 5 min. White Plexiglas was used as the material for the open field test (OFT) device after the previously described (Walsh and Cummins 1976). A 35 W light bulb was used as the light source, hanging around 1 m above the background lighting equipment. The mice were taken from their home cages to the testing room, where they were handled by their tails, put in the equipment, and given 20 min to explore the field (Al‐Amin et al. 2015). The equipment was cleaned with 70% ethanol and left to dry after every excursion. Video of the exploration was captured, and time spent in the center zone was measured (Prut and Belzung 2003).

#### 2.2.7 Social Behaviors

To investigate behavioral alteration, mice were observed for 10 min to count the number of allogrooming, anogenital sniffing events, and crawling under (Morakotsriwan et al. 2016).

#### 2.2.8 Social Interaction Test

The test was administered twice for 20 min. In the first experiment, a mouse was placed in the middle of a Plexiglas box with three connecting chambers. It was given the option to engage with either an empty wire cup in one side chamber or a wire cup containing an unfamiliar mouse (Stranger I) in the opposite chamber, which was matched in strain, age, and sex, after 5 min of habituation. Each cup's interaction time was recorded. In session two, a second control mouse (Stranger II) was placed in a wire confinement cup that was identical to the first one in the other side chamber. This mouse was matched for strain, age, and sex. We timed the duration at which people interacted with each cup (Boccella et al. 2020; Srivastava et al. 2020). We assessed the Sociability Index (SI), a mathematical formula created to enable a direct comparison of groups' social behavior. The SI was calculated as follows (Baronio et al. 2015).

$$\begin{array}{ccc} & & \mathrm{SI}=\\ & & \frac{\left(\mathrm{time}\;\mathrm{exploring}\;\mathrm{novel}\;\mathrm{mouse}\;1-\mathrm{time}\;\mathrm{exploring}\;\mathrm{novel}\;\mathrm{object}\right)}{\left(\mathrm{time}\;\mathrm{exploring}\;\mathrm{novel}\;\mathrm{mouse}\;1+\mathrm{time}\;\mathrm{exploring}\;\mathrm{novel}\;\mathrm{object}\right)} \end{array}$$

### 2.3 Histological Brain Staining

The brains were fixed, cut at 7 µm, and stained with hematoxylin and eosin (H&E) for histological alteration. The slices were then analyzed using an optical microscope using a Leica DM6 microscope (Leica Microsystems Spa, Milan, Italy) (Fusco et al. 2020a, 2020b; Petrosino et al. 2017).

### 2.4 Western Blot Analysis of Cytosolic and Nuclear Extracts

Cytosolic and nuclear extracts were prepared from the right part of the individual brain as previously described (Cordaro et al. 2017; Di Paola, Capparucci, et al. 2021; Di Paola, Iaria, et al. 2021). The following primary antibodies were used: anti‐Inhibitor of Nuclear Factor Kappa‐B alpha (IκB‐α) (1:500; Santa Cruz Biotechnology [SCB], H‐4 sc‐1643), anti‐Nuclear Factor Kappa‐Light‐Chain‐Enhancer of Activated B Cells p65 (NF‐κB p65) (1:500; SCB, F‐6: sc‐8008), anti‐BCL2‐Associated X Protein (Bax) (1:500; SCB, B‐9 sc‐7480), anti‐B‐Cell Lymphoma 2 (Bcl‐2) (1:500; SCB, C‐2 sc‐7382), anti‐Toll‐Like Receptor 4 (TLR4) (1:500; SCB, sc‐293072), anti‐myeloid differentiation primary response 88 (Myd88) (1:500; SCB, sc‐74532), anti‐Glutamate Decarboxylase 65 (GAD65) (1:2000; Abcam), anti‐Glutamate Decarboxylase 67 (GAD67) (1:2000; Abcam), anti‐Glial Fibrillary Acidic Protein (GFAP) (1:500; SCB, sc‐33673) and anti‐Ionized Calcium Binding Adapter Molecule 1 (IBA‐1) (1:500; SCB, sc‐32725) in 1 × PBS, 5% w/v nonfat dried milk, 0.1% Tween‐20 at 4°C overnight (Cordaro, Fusco, et al. 2020; Cordaro et al. 2018; Crupi et al. 2020; Impellizzeri et al. 2016; Paterniti et al. 2017). Blots were further probed with an anti‐β‐actin protein antibody (1:500; SCB) for the cytosolic fraction or an anti‐lamin A/C antibody (1:500; Sigma‐Aldrich Corp., Milan, Italy) for the nuclear fraction to make sure that they were loaded with an equivalent number of proteins (Cordaro, Cuzzocrea, et al. 2020; Di Paola, Impellizzeri, et al. 2016). As directed by the manufacturer, signals were evaluated using an enhanced chemiluminescence (ECL) detection system reagent (Thermo, Monza, Italy). Using BIORAD ChemiDoc TM XRS+ software and densitometry, the relative expression of the protein bands was measured and standardized to the levels of β‐actin and lamin A/C (Di Paola, Cordaro, et al. 2016; Esposito et al. 2016; Paterniti et al. 2015; Peritore et al. 2020; Siracusa et al. 2018).

### 2.5 Biochemical Parameters

The method outlined by was used to determine the level of malondialdehyde, and a spectrophotometer was used to quantify it at 532 nm (Akki et al. 2018). Using TNB (trinitrobenzoic acid)’s molar extinction coefficient, GSH was evaluated using Ellman's method (Ellis 2022; Ellman 2022). The Griess technique was used to estimate the nitrate/nitrite level, and a 540 nm absorbance measurement was taken (Remigante, Spinelli, Straface, et al. 2022; Tsikas 2007). The amount was determined using the potassium nitrite standard curve and given as micromoles of nitrate/nitrite (Remigante, Spinelliet, Pusch, et al. 2022; Tsikas 2005).

### 2.6 Immunohistochemistry of GFAP and IBA‐1

At the end of the experiments, five slices of brain tissue were incubated with anti‐GFAP (1/100 in PBS, SCB) and anti‐IBA‐1 (1/100 in PBS, SCB) as previously described (Fusco et al. 2021; Peritore et al. 2021). After that, sections were treated with peroxidase‐conjugated goat anti‐rabbit IgG or bovine anti‐mouse IgG secondary antibodies (1:2000 Jackson Immuno Research, West Grove, PA, USA). A biotin‐conjugated goat anti‐rabbit IgG or the biotin‐conjugated goat anti‐mouse IgG and avidin‐biotin peroxidase combination (Vector Laboratories, Burlingame, CA, USA) were used to identify specific markers. Using an imaging device (Leica DM6, Milan, Italy), immunohistochemical pictures were captured (LasX Navigator, Milan, Italy). After opening the digital images in ImageJ, they were deconvoluted using the color deconvolution plug‐in. The IHC profiler plug‐in automatically plots the deconvoluted DAB picture into a histogram profile and displays a corresponding scoring log. The computer program returns a positive pixel intensity value that corresponds to the histogram profile. All immunohistochemical investigations were carried out by two researchers who were blind to the treatment (Impellizzeri et al. 2019; Remigante and Morabito 2022; Siracusa et al. 2018).

### 2.7 Cytokine Levels

Commercially available enzyme‐linked immunosorbent assay (ELISA) kits were purchased from R&D Systems (Minneapolis, MN, USA) to estimate the amounts of cytokines IL‐6 and TGF‐β.

### 2.8 Materials

Unless otherwise stated, all compounds were purchased from Sigma‐Aldrich.

### 2.9 Statistical Evaluation

The data in this study are presented as the average ± SEM and represent at least three experiments conducted on various days. *N* denotes the number of animals utilized in in vivo experiments. For each experiment, six brains were used for each technique, and for the histological study, five fields of each brain were examined. The G*Power 3.1 software (Die Heinrich Heine Universität Düsseldorf, Düsseldorf, Germany) was employed to calculate the number of animals used in in vivo research. A competent histopathologist examined the data, without knowledge of the treatment. One‐way ANOVA or two‐way ANOVA was used to examine the data, and then a Bonferroni post hoc test for multiple comparisons was used. A *p* value of 0.05 or less was regarded as significant.

## 3 Results

### 3.1 Behavioral Analysis of CV Administration in VPA‐Mice

**FIGURE 1:**
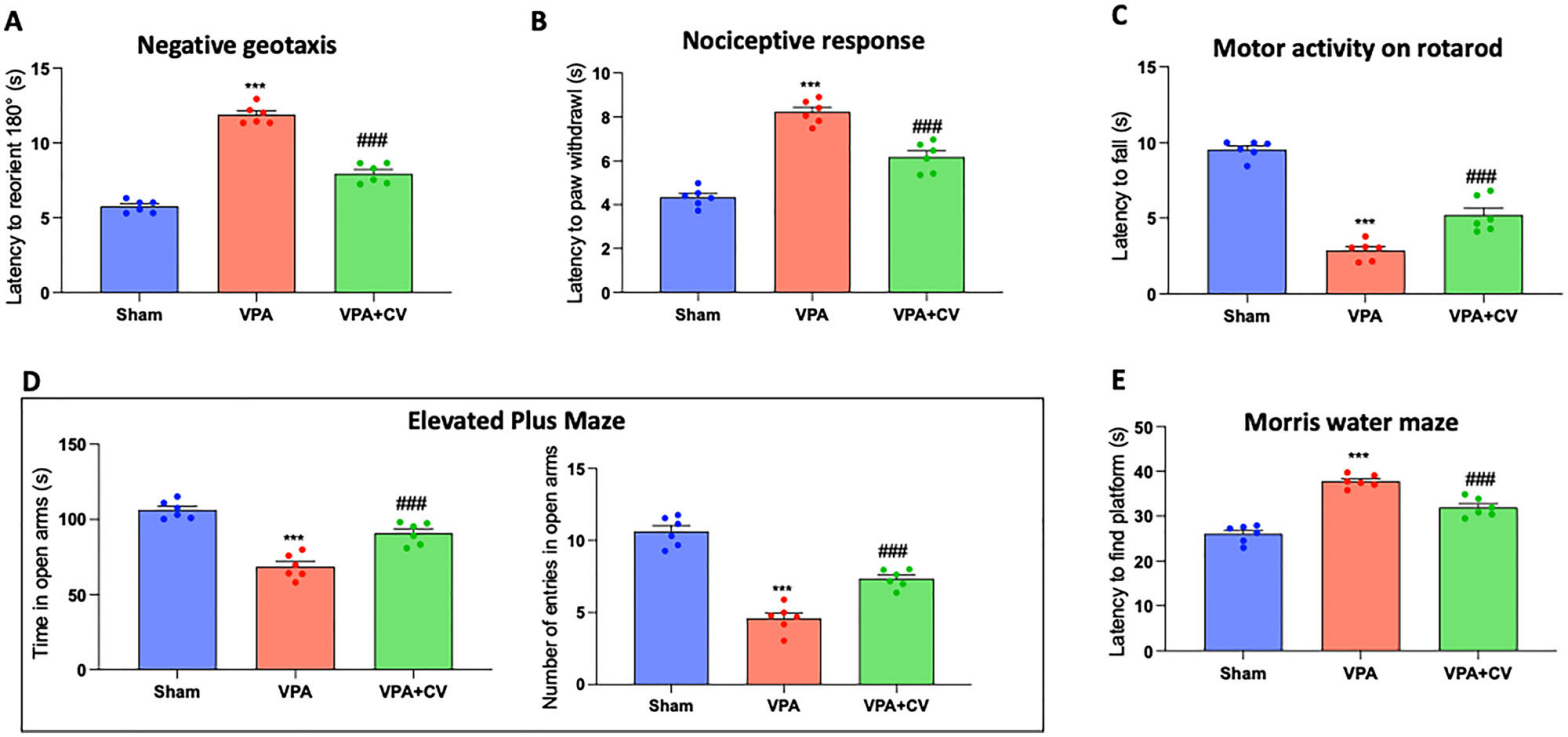
Behavioral analysis of CV administration in VPA‐mice. Negative geotaxis (A); nociceptive test (B); rotarod test (C); elevated plus maze test (D); Morris water maze test (E). ***p < 0.001 versus sham; ^###^ *p* < 0.001 versus VPA; negative geotaxis *F* value: 170.8; nociceptive test *F* value: 73.45; rotarod test *F* value: 97.75; elevated plus maze test *F* value for the time spent in open arms: 40.97; elevated plus maze test *F* value for the number of entries in open arms: 70.13; Morris water maze test *F* value is 60.50. The data are shown as mean ± SEM for each group of six mice. One‐way ANOVA was used to examine the data, and then a Bonferroni post hoc test for multiple comparisons was used.

Compared with the control, VPA administration on PND 14 considerably extended the amount of time (sec) needed to reorient. Compared with the disease control group, treatment with CV (200 mg/kg) has demonstrated a considerable protective effect (Figure 1A). The duration of paw withdrawal in seconds was used as the nociceptive index. Additionally, when compared to the control, mouse pups treated with VPA had a much longer delay in removing their hind paw. Moreover, compared with the disease control, a daily dose of CV (200 mg/kg) had a considerable protective benefit (Figure 1B). The VPA group showed an increase in falling from the rotarod compared with the control group. On the other hand, after daily administration of CV (200 mg/kg), animals took a longer time to fall off rotating rods (Figure 1C). Compared to the control, VPA‐treated mouse pups spent less time in the raised plus maze's open arms. Compared with the disease control, treatment with CV (200 mg/kg) had a significantly higher frequency of entrances and length of time spent in open arms (Figure 1D). Furthermore, when compared to control animals, VPA‐treated mice showed a substantial impairment in escape latency. Compared with the disease control, treatment with CV (200 mg/kg) demonstrated a significantly lower latency to discover the platform (Figure 1E).

### 3.2 CV Administration in VPA‐Mice Ameliorates Social Behaviors

**FIGURE 2:**
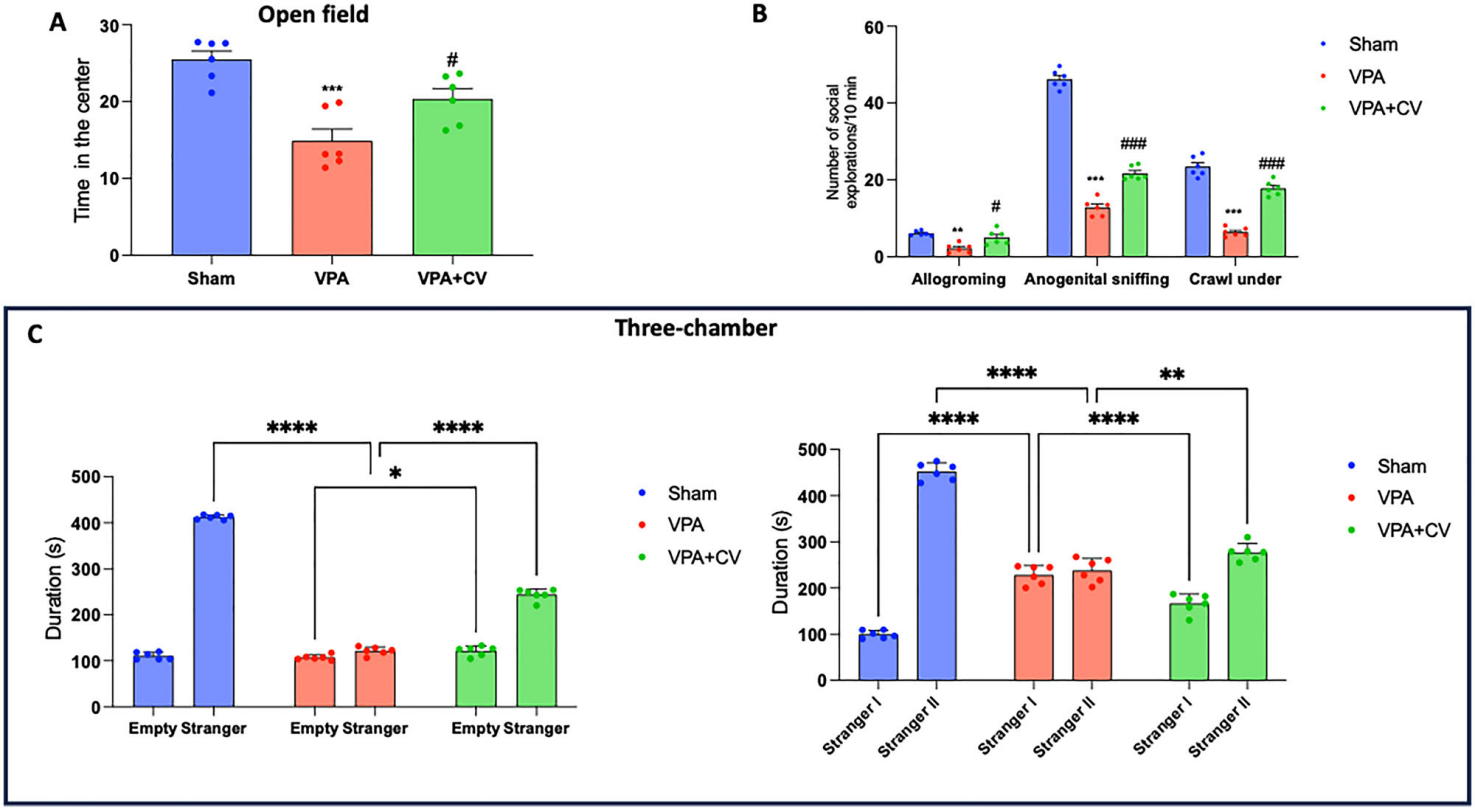
CV administration in VPA‐mice ameliorates social behaviors. Open field test (A); social behaviors (B); three‐chamber test (C). *p < 0.05 versus sham; **p < 0.01 versus sham; ***p < 0.001 versus sham; ^#^ *p* < 0.05 versus VPA; ^###^ *p* < 0.001 versus VPA; Open field test *F* value: 16.00; social behavior *F* value: interaction (116.7), row factor (675.6), column factor (439.8); three‐chamber test *F* value: interaction (815.1), row factor (2474), column factor (837.2); sociability index *F* value: 35.78. The data are shown as mean ± SEM for each group of six mice. One‐way ANOVA (or two‐way ANOVA for social interactions) was used to examine the data, and then a Bonferroni post hoc test for multiple comparisons was used.

According to the results of the OFT, there were considerably fewer mouse crossings in the VPA group than in the control group. In contrast to VPA groups, the number of crossed slots was only slightly higher in the VPA + CV group (Figure 2A). Compared with control mice, the quantity of social explorations (anogenital sniffing, grooming, and crawling under) was dramatically reduced when autism was induced. Following CV (200 mg/kg) therapy, social interactions significantly increased, and conduct returned to normal (Figure 2B). Additionally, in session one of the three‐chamber test, the subject mice in the control group spent substantially more time interacting with other mice than they did in the empty chamber. However, mice in the VPA group did not exhibit a preference for social proximity, as they essentially spent the same amount of time in both the empty and stranger chambers. The amount of time spent with the unfamiliar mouse was significantly longer in the VPA + CV group than it was in the empty chamber. The control group spent considerably more time with Stranger II in session two than they did with Stranger I. However, mice in the VPA group did not exhibit a preference for social proximity since they spent approximately the same amount of time in the chambers containing Strangers I and II. Compared with the time spent in the Stranger I chamber, the time spent with Stranger II in the VPA + CV group was significantly longer (Figure 2C).

### 3.3 CV Dietary Supplements Ameliorate Brain Alteration VPA‐Induced

**FIGURE 3:**
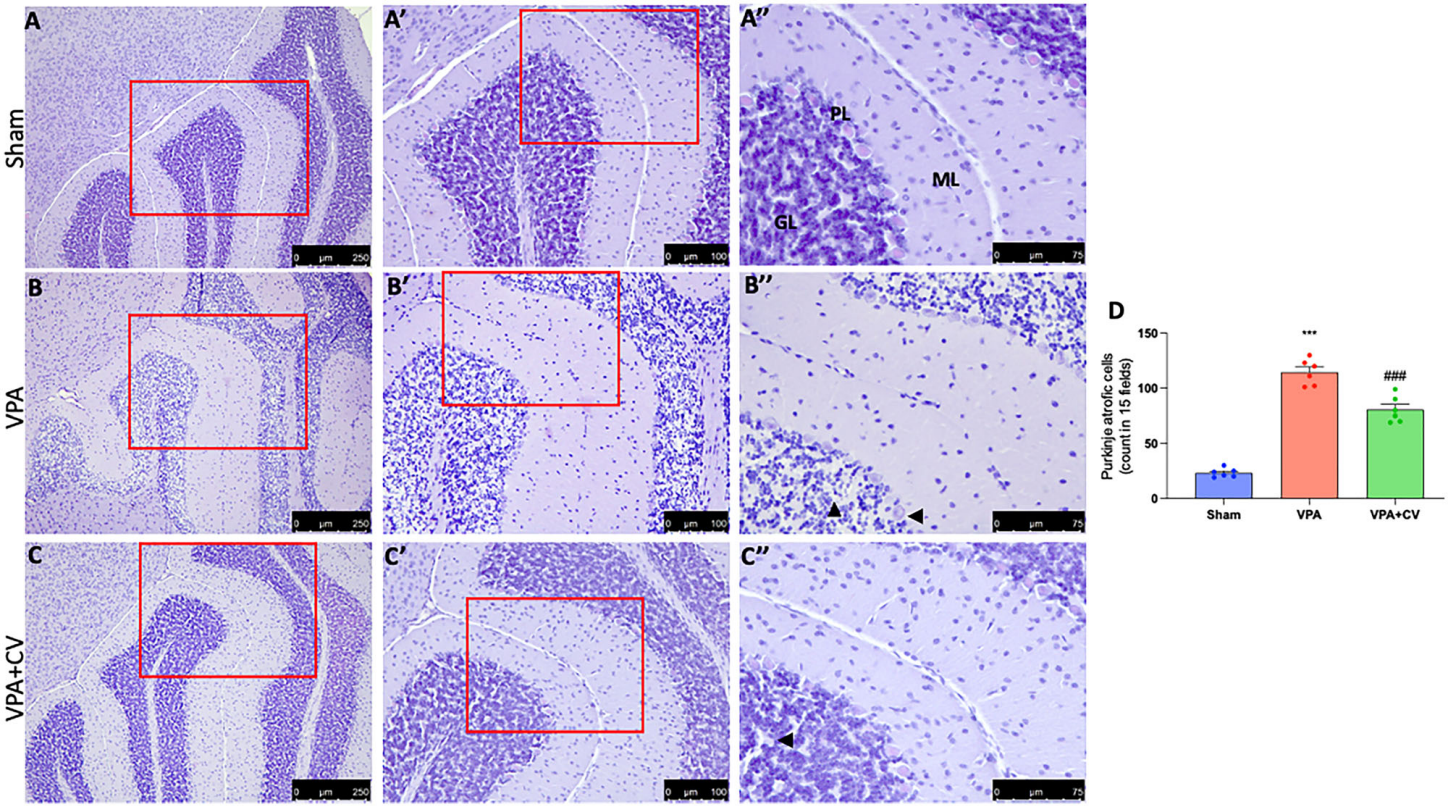
CV dietary supplements ameliorate VPA‐induced cerebellar alteration. Histological photo of Sham (magnification 10× for A, 20× for Aʹ, 40× for Aʺ), VPA (magnification 10× for B, 20× for Bʹ, 40× for Bʺ), and VPA + CV (magnification 10× for C, 20× for Cʹ, 40× for Cʺ); graph (D). Mice given VPA showed a visibly disrupted, chaotic linear organization with pericellular haloes and vacuolated areas in the molecular layer, which matched the morphological changes in the Purkinje layer compared to the sham group. After CV administration, the number of PCs was upregulated in contrast to the VPA group. The granular cell layer contained cerebellar islands that appeared to be normal, but only a few cells displayed pyknosis. ***p < 0.001 versus sham; ^###^ *p* < 0.001 versus VPA; *F* value 129.5. The data are shown as mean ± SEM for each group of six mice. One‐way ANOVA was used to examine the data, and then a Bonferroni post hoc test for multiple comparisons was used.

**FIGURE 4:**
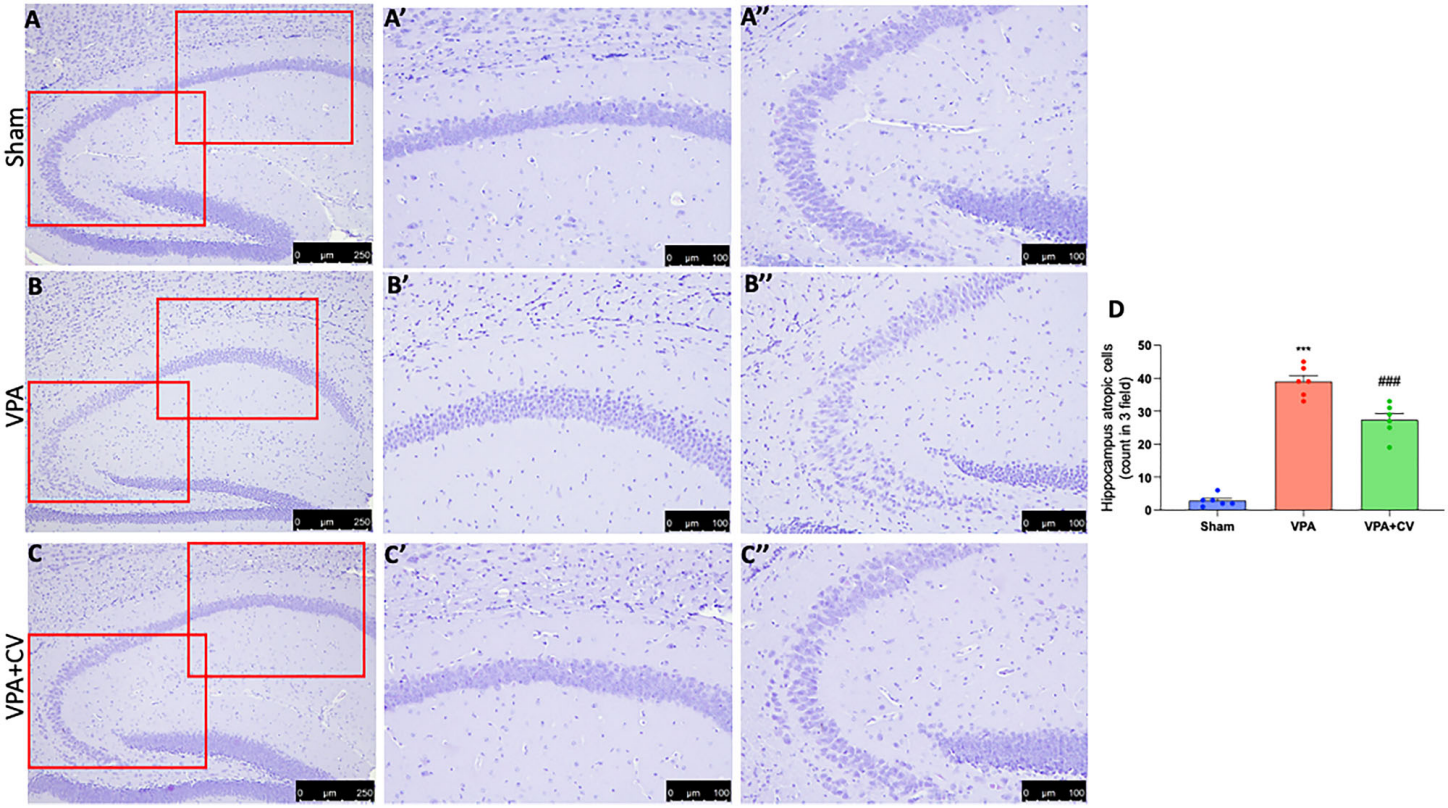
CV dietary supplements ameliorate the VPA‐induced hippocampal alteration. Histological photo of Sham (magnification 10× for [A], magnification 20× for CA1 [Aʹ] and for CA3 [Aʺ]), VPA (magnification 10× for [B], magnification 20× for CA1 [Bʹ] and for CA3 [Bʺ]), and VPA+CV (magnification 10× for [C], magnification 20× for CA1 [Cʹ] and for CA3 [Cʺ]); graph (D). In CA1 and CA3 of the hippocampus, stained with H&E, VPA injection showed more darkly stained degenerated pyramidal neurons and a considerable decline in the mean number of viable neurons in the CA1 and CA3 pyramidal cells of the hippocampus compared to the sham group. CV daily administration significantly improves VPA‐induced neuronal degeneration. ***p < 0.001 versus sham; ^###^ *p* < 0.001 versus VPA; *F* value 126.6. The data are shown as mean ± SEM for each group of six mice. One‐way ANOVA was used to examine the data, and then a Bonferroni post hoc test for multiple comparisons was used.

The molecular layer of the cerebellum of control mice is primarily composed of fibers with a few glial cells. The PCs had conspicuous nucleoli and well‐defined, spherical, vesicular nuclei that were organized in a single row. They have basophilic granules and pale acidophilic cytoplasm. The granular layer was densely packed with spherical cell clumps that were clearly defined, had highly colored nuclei, and had sparse, acidophilic cytoplasm (Figure 3A; see higher magnification Aʹ and Aʺ and graph D). Mice that have been given VPA displayed extensive neuronal affection, particularly in the PC layer. It showed a clearly disorganized regular linear organization that was interrupted. They had unstained and shrinking pericellular halos. These cells had irregular, darkly pigmented nuclei with eosinophilic homogenization in their cytoplasm. Pyknosis was also visible in several nuclei. In comparison to the control group, there were significantly fewer PCs. The Purkinje layer's morphological changes were paralleled by vacuolated regions in the molecular layer. The nuclei of the granular cell layer had pyknosis and were surrounded by unstained pericellular haloes (Figure 3B; see higher magnification Bʹ and Bʺ and graph D). A few impacted PCs were visible in the VPA + CV group alongside the regular ones. Pyknosis was visible in the impacted cells. In comparison to the VPA group, the number of PCs was upregulated. Cerebellar islands that appeared to be normal were present in the granular cell layer, although few cells revealed pyknosis (Figure 3C; see higher magnification Cʹ and Cʺ and graph D). VPA injection exhibited neurodegenerative changes in H&E‐stained sections of the hippocampus area CA1 and CA3 with a significant decrease in the mean number of pyramidal cells as well as in the histological architecture (Figure 4B and higher magnification Bʹ and Bʺ and graph D). They appear as more darkly stained, degenerated pyramidal neurons with a significant decrease in the mean number of viable neurons in hippocampus CA1 and CA3 pyramidal cells, indicating neuron loss compared to the sham group (Figure 4A and higher magnification Aʹ and Aʺ and graph D). Daily CV administration significantly ameliorates the alteration induced by VPA (Figure 4C and higher magnification Cʹ and Cʺ and graph D).

### 3.4 CV Dietary Supplement Attenuates Neuroinflammation

**FIGURE 5:**
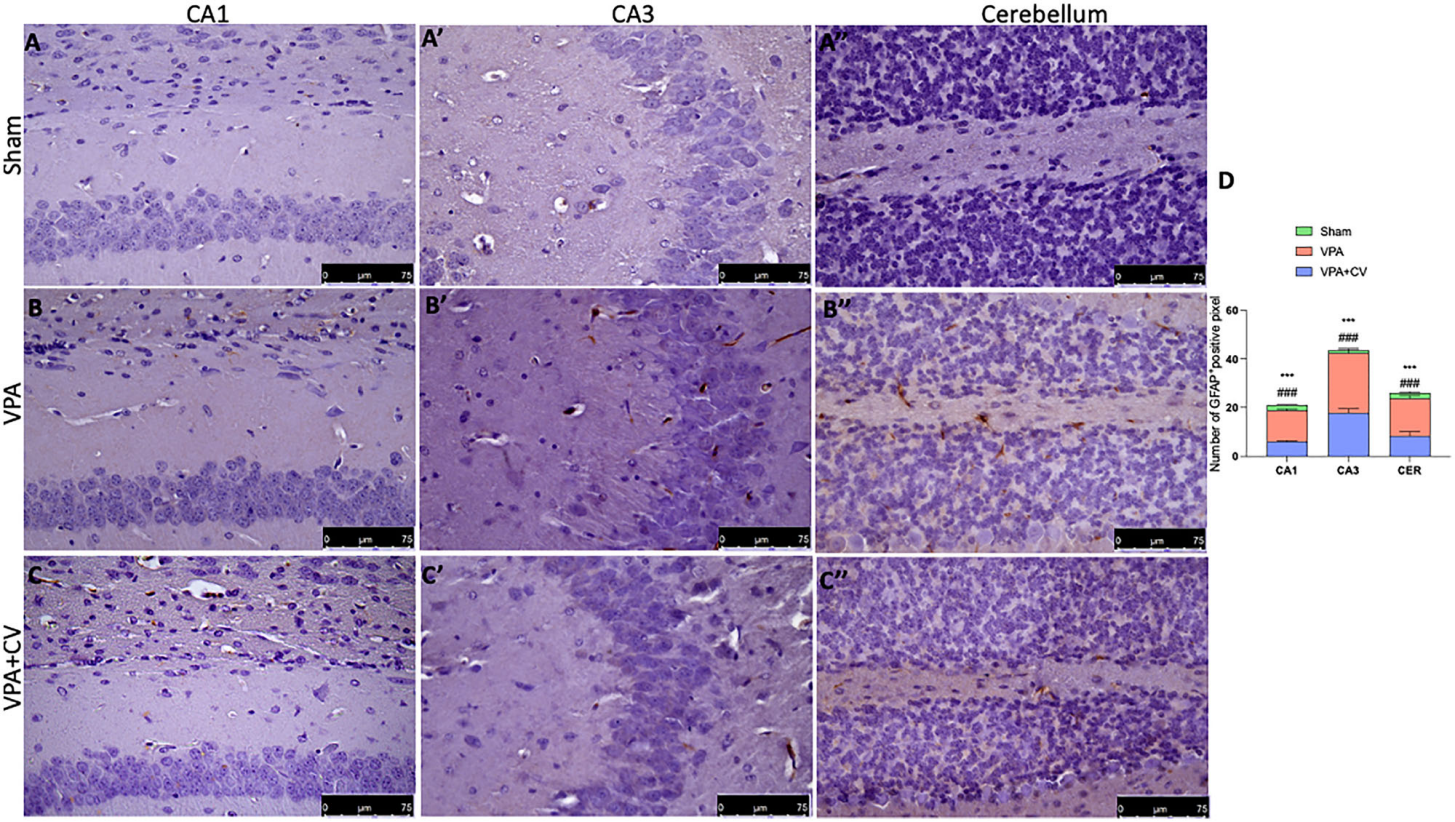
CV daily administration reduced GFAP activation. Immunohistochemistry of GFAP for sham in CA1 (A), CA3 (Aʹ), and cerebellum (Aʺ); VPA in CA1 (B), CA3 (Bʹ), and cerebellum (Bʺ); and VAP + CV in CA1 (C), CA3 (Cʹ), and cerebellum (Cʺ). Graphical representation of positive pixels (D). ***p < 0.001 versus sham; ^###^ *p* < 0.001 versus VPA; *F* value: interaction (11.49); row factor (34.55); column factor (136.3). The data are shown as mean ± SEM for each group of six mice. One‐way ANOVA was used to examine the data, and then a Bonferroni post hoc test for multiple comparisons was used.

**FIGURE 6:**
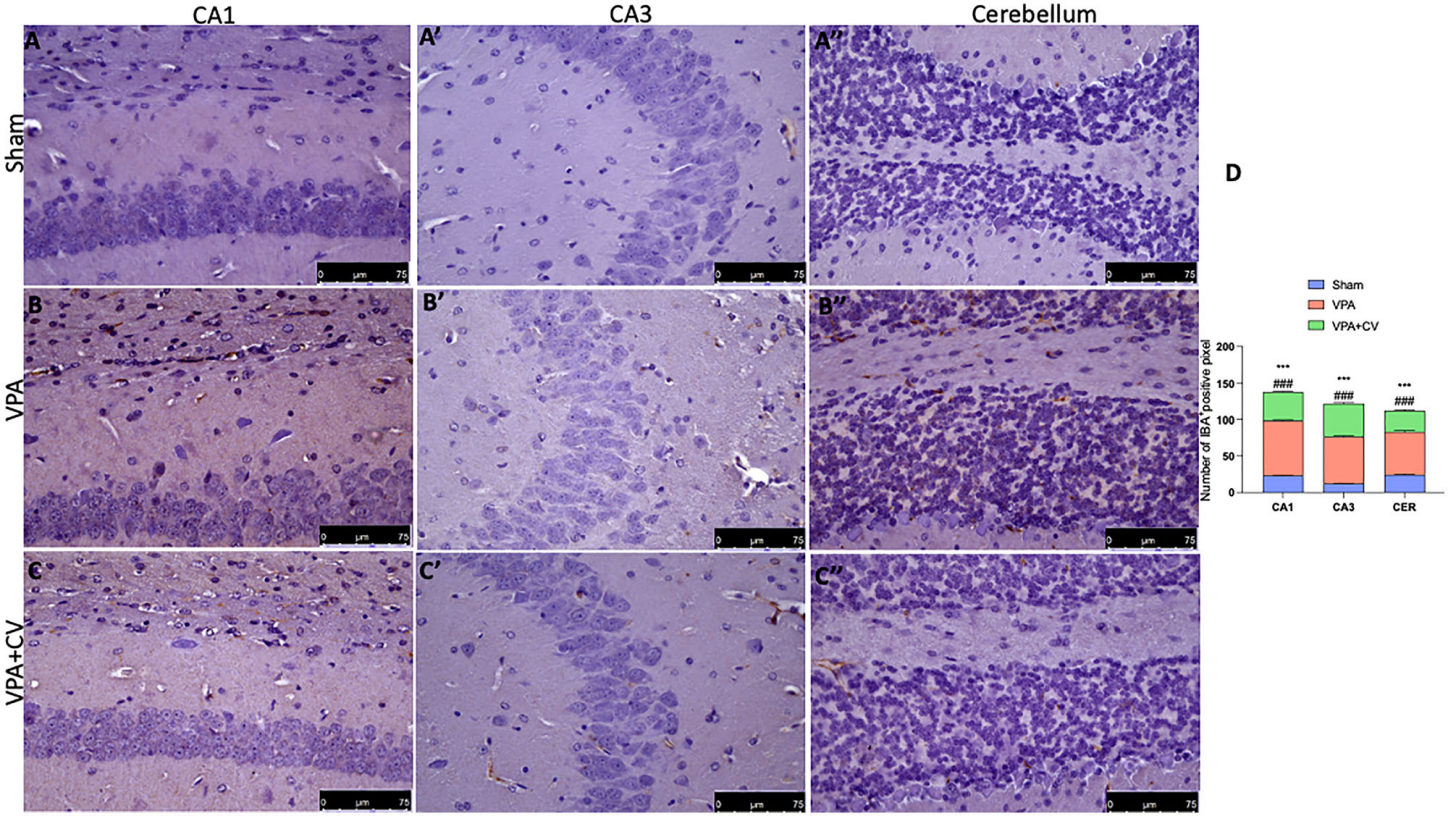
CV daily administration reduced IBA‐1 activation. Immunohistochemistry of IBA‐1 for sham in CA1 (A), CA3 (Aʹ), and cerebellum (Aʺ); VPA in CA1 (B), CA3 (Bʹ), and cerebellum (Bʺ); and VAP + CV in CA1 (C), CA3 (Cʹ), and cerebellum (Cʺ). Graphical representation of positive pixels (D). ***p < 0.001 versus sham; ^###^ *p* < 0.001 versus VPA; *F* value: interaction (42.93); row factor (37.42); column factor (1107). The data are shown as mean ± SEM for each group of six mice. One‐way ANOVA was used to examine the data, and then a Bonferroni post hoc test for multiple comparisons was used.

**FIGURE 7:**
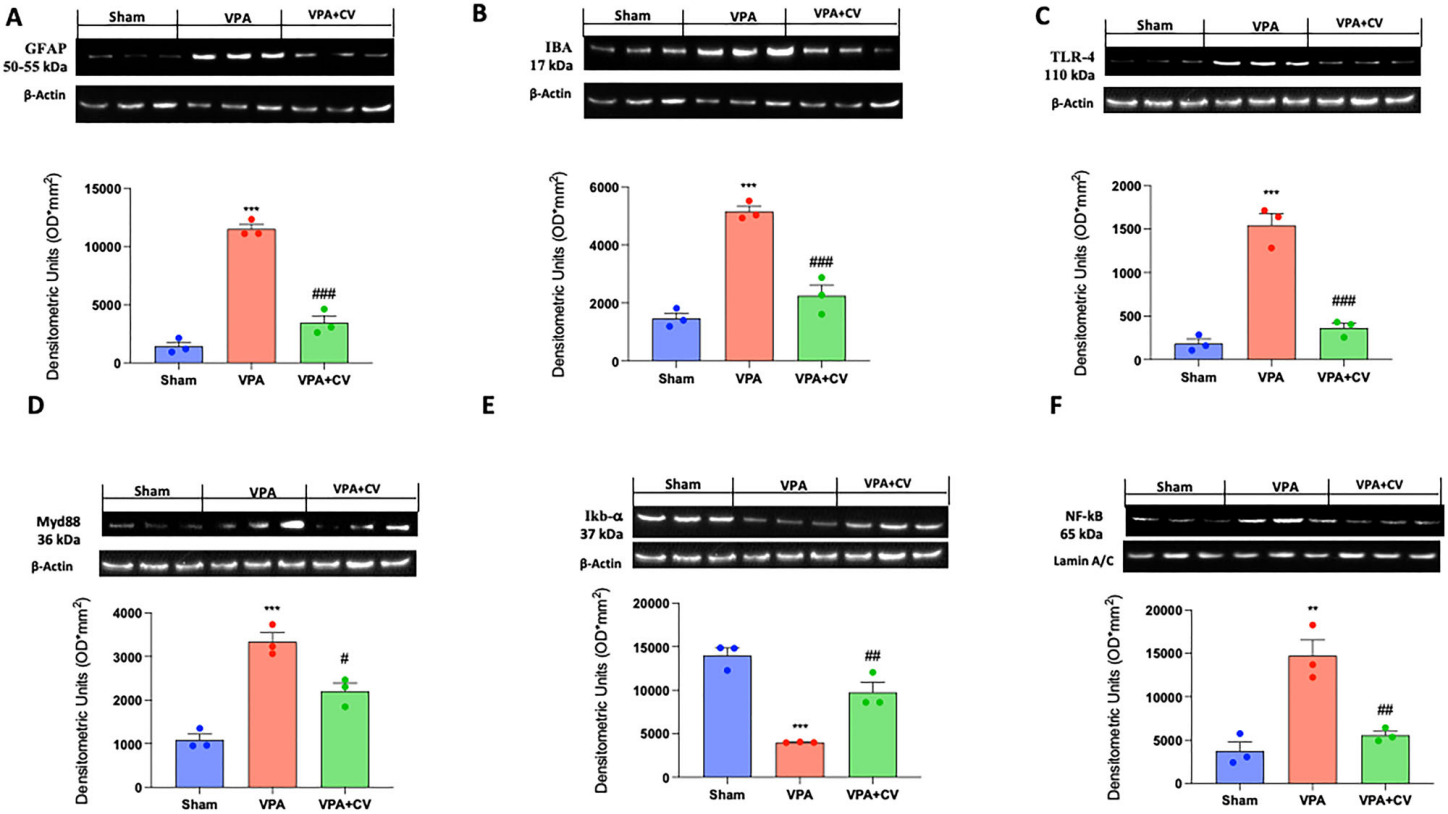
CV dietary supplements modulate TLR‐4/MYD‐88/NF‐κB pathways. Western blots and relative densitometric analysis of GFAP (A), IBA‐1 (C), TLR‐4 (C), MYD‐88 (D), IκB‐α (E), and NF‐κB (F). **p < 0.01 versus sham; ***p < 0.001 versus sham; ^#^ *p* < 0.05 versus VPA; ^##^ *p* < 0.01 versus VPA; ^###^ *p* < 0.001 versus VPA. GFAP *F* value 127.1; IBA‐1 *F* value 56.47; TLR‐4 *F* value 70.15; MYD‐88 *F* value 41.44; IκB‐α *F* value 50.38; NF‐κB *F* value 22.86. The data are shown as mean ± SEM for each group of six mice. One‐way ANOVA was used to examine the data, and then a Bonferroni post hoc test for multiple comparisons was used.

Microglia and astrocyte function in ASD has attracted increasing attention in recent years. For this reason, we first investigated with immunochemistry and then, by Western blot, two specific cell markers, GFAP and IBA‐1. We found that in the group subjected to VPA injection, there was a significant increase in CA1, CA3, and cerebellum in GFAP (Figure 5B,Bʹ, and Bʺ; see graph of positive pixels D) and IBA‐1 (Figure 6B,Bʹ, and Bʺ; see graph of positive pixels D) compared to the sham group (Figure 5A,Aʹ, and Aʺ; see graph of positive pixels D for GFAP and Figure 6A,Aʹ, and Aʺ; see graph of positive pixels D for IBA‐1). The same trends were also observed by Western blots in the whole brain homogenates (Figure 7A and relative densitometric analysis for GFAP and Figure 7B and relative densitometric analysis for IBA‐1). Daily administration of CV significantly decreases the number of positive cells in all brain areas considered (Figure 5C,Cʹ, and Cʺ; see graph of positive pixels D for GFAP and Figure 6C,Cʹ, and Cʺ; see graph of positive pixels D for IBA‐1) as well as in the whole brain (Figure 7A and relative densitometric analysis for GFAP and Figure 7B and relative densitometric analysis for IBA‐1).

### 3.5 CV Dietary Supplement Modulates TLR‐4/MYD‐88/NF‐κB Pathways

TLR4, which is expressed on both innate and adaptive cells, including T cells and B cells, particularly during an inflammatory state, has also been proven to play a significant role in neuroinflammation. T cells and monocytes from people with ASD have also been found to have elevated TLR4‐related immunological responses. The functional importance of TLR4 signaling is determined by its relationship to inflammatory mediators and oxidative stress in various immune cells (Al‐Harbi et al. 2020). For this reason, we investigated these pathways using Western blots. We found that in the group that received VPA injection, there was a significant activation of TLR‐4 (Figure 7C and relative densitometric analysis), as well as of MYD‐88 (Figure 7D and relative densitometric analysis) and NF‐κB (Figure 7F and relative densitometric analysis), with a significant decrease of IκB‐α (Figure 7E and relative densitometric analysis) compared to the sham group. The daily administration of CV not only improved the activation of the TLR4 pathway but also brought it back to almost physiological conditions.

### 3.6 CV Dietary Supplement Improves GABA System

**FIGURE 8:**
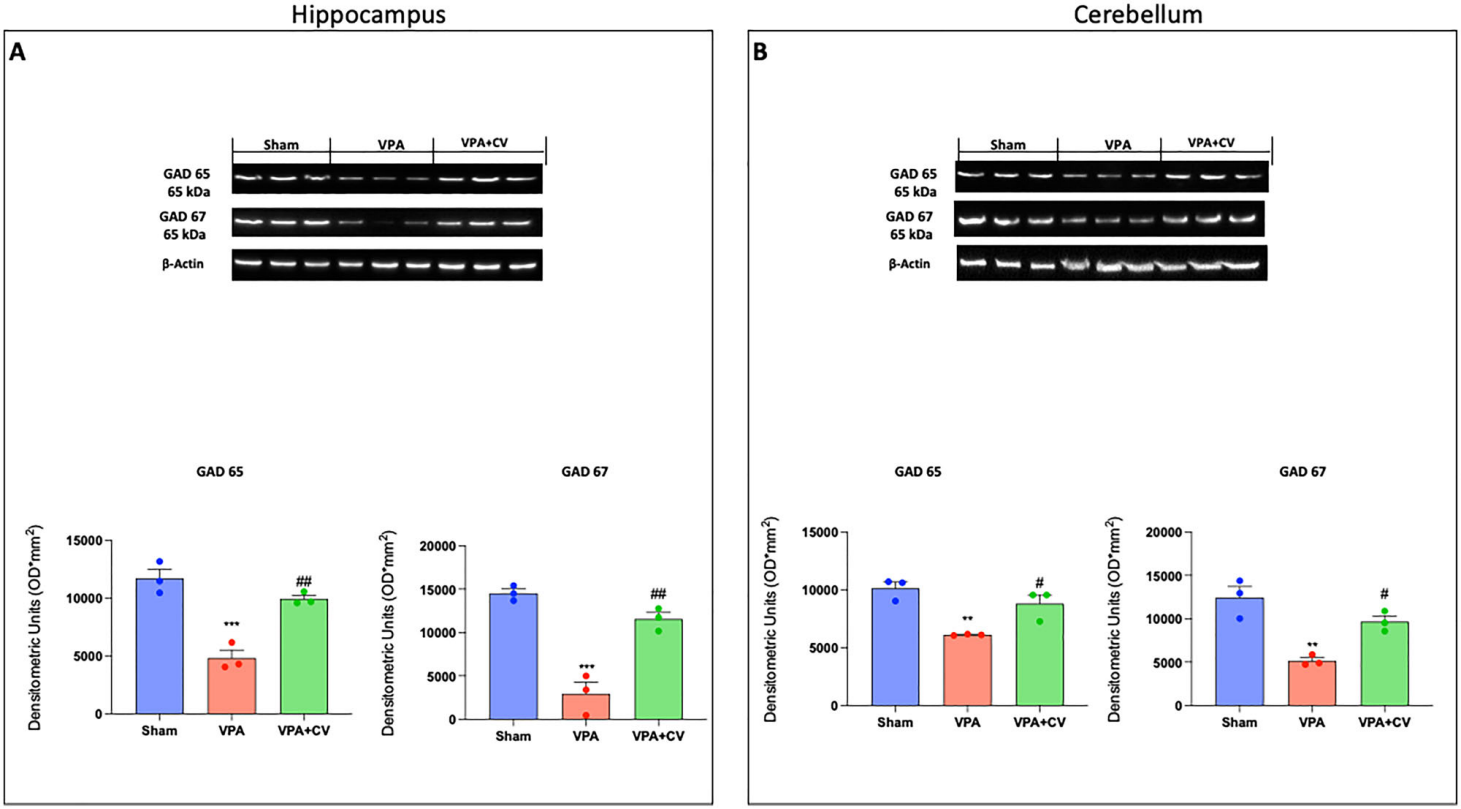
CV dietary supplements improve the GABA system. Western blots and relative densitometric analysis of GAD 65 and GAD 67 in the hippocampus (A). Western blots and relative densitometric analysis of GAD 65 and GAD 67 in the cerebellum (B). **p < 0.01 versus sham; ***p < 0.001 versus sham; ^#^ *p* < 0.05 versus VPA; ^##^ *p* < 0.01 versus VPA; GAD 65 *F* value is 32.54 and GAD 67 *F* value is 41.96 in the hippocampus. GAD 65 *F* value is 14.28 and GAD 67 *F* value is 18.29 in the cerebellum. The data are shown as mean ± SEM for each group of six mice. One‐way ANOVA was used to examine the data, and then a Bonferroni post hoc test for multiple comparisons was used.

The glutamate–glutamine cycle is a crucial mechanism for maintaining the homeostasis of GABA in ASD. We looked at the glutamic acid decarboxylase (GAD) expression in the hippocampus and cerebellum because glutamate in GABAergic neurons is decarboxylated to form GABA. We found that there was a significant decrease in GAD 65 and GAD 67 in both the hippocampus (Figure 8A) and cerebellum (Figure 8B) compared to the sham group. On the other hand, after daily administration of 200 mg/kg of CV, there is a significant restoration in both expressions.

### 3.7 CV Daily Administration Ameliorates Biochemical Parameters

**FIGURE 9:**
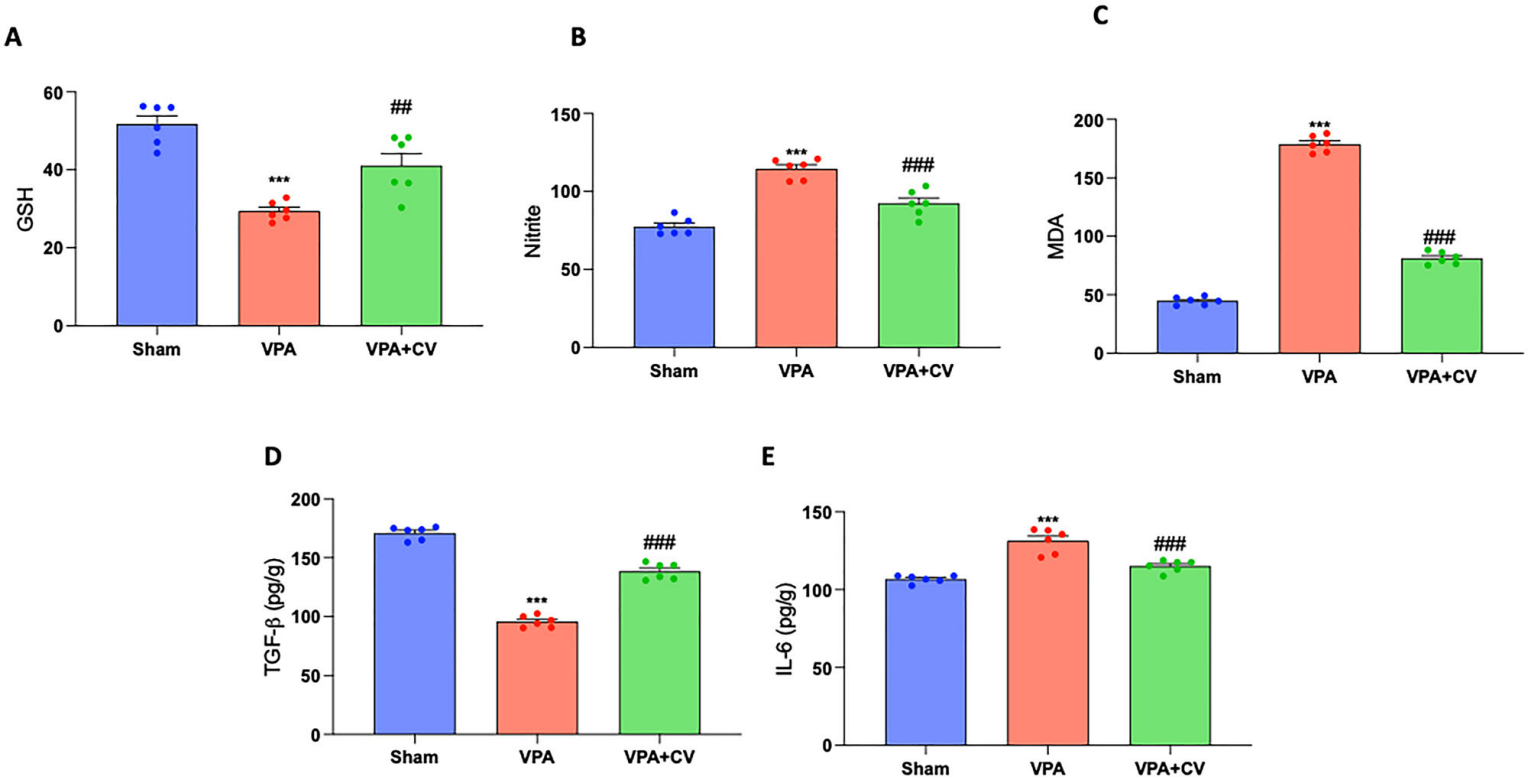
CV daily administration ameliorates biochemical parameters. Brain analysis of GSH (A), nitrite (B), MDA (C), TGF‐b (D), and IL‐6 (E). ***p < 0.001 versus sham; ^##^ *p* < 0.01 versus VPA; ^###^ *p* < 0.001 versus VPA. GSH *F* value 24.97; nitrite *F* value 44.37; MDA *F* value 967.3; TGF‐β 245.7; IL‐6 *F* value 34.64. The data are shown as mean ± SEM for each group of six mice. One‐way ANOVA was used to examine the data, and then a Bonferroni post hoc test for multiple comparisons was used.

VPA‐treated animals showed a significant decrease in glutathione levels (Figure 9A) and TGF‐β (Figure 9D), as well as a considerable increase in total nitrite levels (Figure 9B), MDA (Figure 9C), and IL‐6 (Figure 9E) compared to the sham group. All parameters considered were restored to values that were nearly physiological with the daily administration of CV at a dose of 200 mg/kg.

## 4 Discussion

ASD is a collection of various neurodevelopmental conditions characterized by early‐onset impairment in social cognition and social perception, executive dysfunction, unusually constrained, repetitive behaviors and interests, and abnormal perceptual and information processing issues in social communication (Bertolino et al. 2017). Humans exposed to VPA in utero exhibit signs comparable to autism, including delayed language and communication development, stereotypical behaviors, hyperexcitability, and behavioral developmental delays (Williams et al. 2001). VPA exposure throughout pregnancy and/or the early postnatal period caused neurodevelopmental impairments similar to the motor and cognitive changes seen in autistic people (Markram et al. 2008; Wagner et al. 2006). On P14, crucial cerebellar‐mediated behaviors first became visible (Rice and Barone 2000). A single 400 mg/kg VPA dose on P14 resulted in motor and cognitive abnormalities in our study that mimicked the regression of autism (Rice and Barone 2000). According to different studies, sodium valproate administration on PND 14 significantly increased autistic symptoms (Bertolino et al. 2017; Mony et al. 2016; Norton et al. 2020; Pragnya et al. 2014; Reynolds et al. 2012). These symptoms included behavioral abnormalities such as decreased pain sensitivity, loss of motor skill development (delayed negative geotaxis response), increased locomotor activity in novel environments, decreased social behavior, increased anxiety in elevated plus mazes, and performance delays in water mazes (Banji et al. 2011; Rossignol and Frye 2014). In our work, we found that daily administration of CV at the dose of 200 mg/kg significantly ameliorated behavioral changes induced by VPA. The significant drop (usually 35%–50%) in the number of PCs in the cerebellum of the brain and the disarray of the hippocampus are two of the consistent biological findings of autism. PCs, which are the cerebellum's output neurons, integrate complicated information before projecting to the rest of the brain (Pragnya et al. 2014). The behavioral symptoms of autism spectrum diseases are indicative of decreased PC numbers, which are believed to mediate aberrant activity. In our work, histological analyses revealed that sodium valproate injection induced myelin loss, PC layer damage, cerebellar granule cell loss, and disarray in CA1 and CA3 hippocampi (Thomas et al. 1998). Following daily oral treatment of CV at a dose of 200 mg/kg, the integrity of the hippocampus and PC layer was recovered. Over the past four decades, numerous investigations have shown that people with ASD have altered neuroinflammation with a significant activation in astrocytes and microglia (Eissa et al. 2020). In our work, we found that VPA injection induced an increase in GFAP and IBA‐1 expression not only in the hippocampus and cerebellum but also in the whole brain. Astrogliosis and microgliosis were significantly attenuated by daily administration of 200 mg/kg of CV. TLR4 signaling has been implicated in several immune and neuropsychiatric illnesses, including anxiety, autism spectrum disorder, psoriasis, and multiple sclerosis. In brain injuries, TLR4 has been shown to trigger the production of cytokines and microglial activation. Microglial activation brought on by neuroinflammation damages the growing brain. Inflammatory mediators produced by microglia might be involved in this process (Yao et al. 2013). TLR4 activation in B cells is linked to NF‐κB, which can induce a variety of inflammatory and oxidative mediators in these cells (Rose et al. 2018). Al‐Harbi et al. (2020) showed that increased NF‐κB expression is associated with increased TLR4 expression on B cells. These results could have implications for both systemic and neuroinflammation. It is generally recognized that individuals with ASD have a dysregulated gut environment, which causes intestinal barrier function to be disturbed (Shin et al. 2009). According to reports, increased gut permeability can lead to the transmigration of bacterial byproducts that may activate TLR4 in peripheral B cells. As a result of immune cells adhering to the vascular endothelium and infiltrating the neural tissue, ROS generation by neutrophils, T cells, and monocytes is a well‐known essential factor of neuroinflammation. This was demonstrated in an animal study where TLR4 activation during pregnancy resulted in brain developmental abnormalities in the fetus that were thought to be brought on by oxidative stress (Dargenio et al. 2023; Haroun et al. 2023). Myeloid differentiation primary response 88 (MYD88) and interleukin receptor‐associated kinase (IRAK) are two of the proteins that are involved in the complicated intracellular cascade that leads to the activation of NF‐κB in the case of TLR signaling (Heine and Lien 2003; Ishii et al. 2005). In our study, we found that CV possesses the significant ability to restore TLR4/Myd88/NF‐κB pathways almost to physiological levels, as well as the release of TGF‐β and IL‐6. Furthermore, have shown a link between GABA homeostasis and TLR‐4 activation. A glutamate–glutamine cycle occurs between astrocytes and neurons. Glial glutamate transporters transfer extracellular glutamate into an astrocyte. Glutamate synthetase operates inside the astrocyte to convert glutamate to glutamine. The glutamine is subsequently exported by the astrocyte outside the cell, where it is absorbed by neurons. In neurons, phosphate‐activated glutaminase deaminates glutamine to produce glutamate. GAD then decarboxylates glutamate in GABAergic neurons to produce GABA. The cycle is concluded when vesicular GABA transporters transfer GABA into synaptic vesicles (Yan et al. 2015). In our work, we demonstrated that following the daily administration of CV, there was an increase in the expression of GAD, which was reduced by the administration of VPA, bringing it back almost to physiological levels. Studies in both the clinical and preclinical stages showed that oxidative stress was present, as shown by an increase in lipid peroxidation and a decrease in glutathione. In the current investigation, we found that total nitrite and MDA levels increased while glutathione levels fell, supporting the notion that oxidative stress plays a role in sodium valproate‐induced autism (Pragnya et al. 2014). A negative loop occurs as a result of low glutathione levels at the time of VPA exposure, increasing the need for glutathione and inhibiting glutathione synthesis for an extended length of time (Kern and Jones 2006).

As highlighted in the literature, there are several experimental models that reproduce the core ASD‐like behaviors (social communication deficits and repetitive behaviors) and some comorbid features, including anxious behavior, motor deficits, and abnormal sensory processing. Among the most widely used are prenatal exposure to valproic acid and knockout models. However, it is not always possible to use these models in research. Although in our study, we focused on postnatal exposure to VPA, which not only produces deficits in core ASD‐like behaviors (social interaction and repetitive‐stereotyped behaviors) but also leads to one or more comorbid features, including altered sensory processing, anxious behavior, and disturbed motor functions, readers should note the use of this model as a possible limitation of this work.

## 5 Conclusions

These results led us to the conclusion that daily CV administration, through its cognition‐enhancing, anti‐inflammatory, and neuroprotective activity, ameliorates sodium valproate‐induced behavioral deficits, neuroinflammation, oxidative stress markers, and GABAergic system via the TLR‐4/Myd88/NF‐κB signaling cascade. We want to be clear that the complexity and significant variety of the clinical manifestations of ASD warrant certain limitations in this investigation.

## Author Contributions

**Francesco Molinari**: methodology. **Roberta Fusco**: investigation. **Rosalba Siracusa**: investigation. **Ramona D'amico**: investigation. **Daniela Impellizzeri**: investigation. **Ali S. Abdelhameed**: formal analysis. **Tilman Fritsch**: formal analysis. **Ursula M. Jacob**: formal analysis. **Salvatore Cuzzocrea**: resources. **Vittorio Calabrese**: resources. **Rosanna Di Paola**: supervision, project administration. **Marika Cordaro**: conceptualization, writing – review and editing.

## Conflicts of Interest

The authors declare no conflicts of interest.

### Peer Review

The peer review history for this article is available at https://publons.com/publon/10.1002/brb3.70591

## Supporting information



Figure S1. Preliminary behavioral evaluation. Negative geotaxis (A); nociceptive test (B); rotarod test (C); elevated plus maze test (D); Morris water maze test (E). No significant difference was found between the sham and sham + CV groups. ***p < 0.001 versus sham; ^###^ *p* < 0.001 versus VPA. The data are shown as mean ± SEM for each group of six mice. One‐way ANOVA was used to examine the data, and then a Bonferroni post hoc test for multiple comparisons was used.

## Data Availability

The datasets generated and/or analyzed for the present study are available from the corresponding author on reasonable request.
