# Supplementary figures and images for: ⁠⁠Functional Foods Alleviate Behavioral Alterations and Improve GABAergic System Regulating TLR‐4/NF‐κB Axis in Valproic‐Induced Autism
Source: Brain Behav. 2025 Jun 4;15(6):e70591. doi: 10.1002/brb3.70591 (PMC12134490; doi:10.1002/brb3.70591)

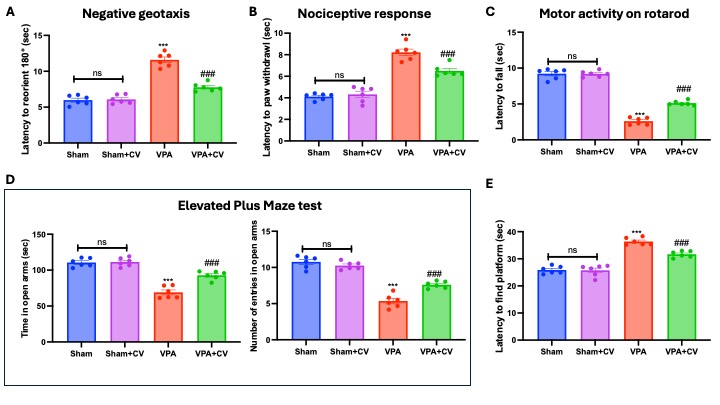

Supplement: Supplementary file 1 — Figure S1. Preliminary behavioral evaluation. Negative geotaxis (A); nociceptive test (B); rotarod test (C); elevated plus maze test (D); Morris water maze test (E). No significant difference was found between the sham and sham + CV groups. ***p < 0.001 versus sham; ### p < 0.001 versus VPA. The data are shown as mean ± SEM for each group of six mice. One‐way ANOVA was used to examine the data, and then a Bonferroni post hoc test for multiple comparisons was used. [file BRB3-15-e70591-s001.jpeg]
